# Isolation and Characterization of Urease-Producing Soil Bacteria

**DOI:** 10.1155/2021/8888641

**Published:** 2021-07-09

**Authors:** Eshetu Mekonnen, Ameha Kebede, Asefa Nigussie, Gessese Kebede, Mesfin Tafesse

**Affiliations:** ^1^Dire Dawa University, College of Natural and Computational Sciences, Department of Biology, Dire Dawa, Ethiopia; ^2^Haramaya University, College of Natural and Computational Sciences, School of Biology and Biotechnology, Haramaya, Ethiopia; ^3^Addis Ababa Science and Technology University, Department of Biotechnology, Addis Ababa, Ethiopia

## Abstract

Urease is an enzyme produced by ureolytic microorganisms which hydrolyzes urea into ammonia and carbon dioxide. Microbial urease has wide applications in biotechnology, agriculture, medicine, construction, and geotechnical engineering. Urease-producing microbes can be isolated from different ecosystems such as soil, oceans, and various geological formations. The aim of this study was to isolate and characterize rapid urease-producing bacteria from Ethiopian soils. Using qualitative urease activity assay, twenty urease-producing bacterial isolates were screened and selected. Among these, three expressed urease at high rates as determined by a conductivity assay. The isolates were further characterized with respect to their biochemical, morphological, molecular, and exoenzyme profile characteristics. The active urease-producing bacterial isolates were found to be nonhalophilic to slightly halophilic neutrophiles and aerobic mesophiles with a range of tolerance towards pH (4.0–10.0), NaCl (0.25—5%), and temperature (20–40°C). According to the API ZYM assays, all three isolates were positive for alkaline phosphatase, leucine aryl amidase, acid phosphatase, and naphthol_AS_BI_phosphohydrolase. The closest described relatives of the selected three isolates (Isolate_3, Isolate_7, and Isolate_11) were *Bacillus paramycoides*, *Citrobacter sedlakii*, and *Enterobacter bugandensis* with 16S rRNA gene sequence identity of 99.0, 99.2, and 98.9%, respectively. From the study, it was concluded that the three strains appear to have a relatively higher potential for urease production and be able to grow under a wider range of growth conditions.

## 1. Introduction

Urease is an enzyme that catalyzes the hydrolysis of urea by all plants and many algae, fungi, and bacteria [1]. As a consequence, urease activity (urea amidohydrolase: EC 3.5.1.5) is widely distributed in soil [2, 3]. Microbial urease has also been studied in clinical samples as it is related to the virulence of pathogenic microorganisms [4], contributing to urinary stones, pyelonephritis, and gastric ulceration [5, 6]. Ureases were immobilized and used as a biosensor in the construction of a flow cell with the incorporation of a urease-modified device for the continuous measurement of urea in flowing systems [7]. They were also used along with urea fertilizer to ease the hydrolysis of ammonium into the soil [8]. However, in the last two decades, the use of microbial urease has switched from clinical relevance to geotechnical engineering and applied biotechnology [9], because of the abilities of microorganisms to induce calcite precipitation, a common natural soil cementing agent, in the presence of urea and calcium ions [10, 11].

Several aerobic bacteria genera (i.e., *Proteus*, *Morganella*, *Serratia*, *Pseudomonas*, *Clostridium*, *Fusobacterium*, *Ureaplasma*, *Providencia*, *Sarcina*, *Lactobacillus*, *Streptococcus*, and *Enterobacter*) are known to produce the enzyme urease and are able to degrade urea in the soil under aerobic conditions [1, 12]. Urease turns the uncharged urea molecule into two charged ions: ammonium (NH^4+^, positively charged) and carbonate (CO_3_^2−^, negatively charged) [4, 12]. As a result, the ammonium (NH^4+^) released from urea hydrolysis results in local pH rise and commences the precipitation of calcium carbonate [13].

Microbial urease can exist in two possible states in soil. It occurs either intracellularly, associated directly with ureolytic microorganisms, or extracellularly, after being released from cells [14, 15]. Urease-producing bacteria are of particular interest for the production of complex bioenzymes and are known to produce other soil enzymes [16] that lead to the stabilization of expansive clays [17] through cation exchange and flocculation of the clay minerals [18, 19].

Reference [20] estimated that the microorganisms capable of hydrolyzing urea comprised between 17 and 30% of the aerophilic, microaerophilic, and anaerobic microorganisms isolated from their soil samples. Their ability to produce urease can be exploited to enrich and isolate such bacteria from the environment for future applications [21]. While the occurrence of these bacteria and their characteristics have been explored in some regions and soil types [1, 12, 21, 22] and other novel bacterial strains isolated from Ethiopian sediments and soils [23, 24], this study is the first report on the characterization of ureolytic bacteria from Ethiopian soil. This study aimed to isolate and characterize rapid urease-producing bacteria from Ethiopian soils. Thus, ureolytic bacteria were isolated from soils and were identified based on their urease activity and 16S rRNA gene sequence analysis. Selected rapid urease producer strains were further characterized by biochemical, morphological, molecular, and exoenzyme profile characteristics.

## 2. Materials and Methods

### 2.1. Soil Sampling

Soil samples were collected from different types of ecosystems including a urea dumping site, stable soil structures such as termite casts, and rift valley soda lakeshores of Ethiopia. The samples were collected in summer 2017 from Tulu Bolo Fertilizer Factory (pH = 8.15, soil temperature = 28°C, 8.6633°N, 38.2164 °E, and at an elevation of 2193 meters above sea level); shore soil of Lake Abijata (pH = 10.5, soil temperature = 32°C, 7.6167°N, 38.6000°E, and at an elevation of 1573 meters above sea level); shore soil of lake Chitu (pH = 11.5, soil temperature = 30°C, 7.403599°N, 38.423527°E, and at an elevation of 1539 meters above sea level); a termite mound in the Wonji area (pH = 7.56, soil temperature = 33°C, 8.450919°N, 39.278972°E, and at an elevation of 1618.28 meters above sea level); termite mounds near the town of Yabello (pH = 7.9, soil temperature = 31°C, 4.889622°N, 38.084775°E, and at an elevation of 1,857 meters above sea level); and a termite mound in West Wollega (pH = 6.7, soil temperature = 30°C, 9.487993°N ,35.526785°E, and at an elevation of 1821 meters above sea level) [25].

The soil samples consisted of homogenized composite samples taken from multiple sample units as described in [26]. The soil samples were collected from the upper 10 cm of the topsoil, sampling was done using a sterile spatula, and the samples were kept in sterile polyethylene bags [27]. The samples were immediately stored in an ice-box at 4°C and transported to the laboratory at Addis Ababa Science and Technology University.

### 2.2. Enrichment and Screening of Ureolytic Bacterial Isolates

To enrich urease-producing bacteria from soil samples, 1 g of each soil sample was inoculated into 100 mL of urea broth medium (Sigma-Aldrich) consisting of 1.00 mg/L peptone, 1.000 mg/L dextrose, 5.00 mg/L sodium chloride, 1.2 mg/L disodium phosphate, 0.8 mg/L monopotassium phosphate, 0.012 mg/L of phenol red, and 6% (w/w) urea (HiMedia, sterile filtered 0.45 *μ*m, added after autoclaving) (in 250 mL shake flasks) and incubated under aerobic batch conditions at 30°C for 120 h under shaking condition at 130 rpm [21]. For subsequent enrichment, 20% (v/v) of the culture samples were intermittently transferred (up to four times) into a fresh medium [28]. For bacterial isolation, an aliquot of 1 mL was serially diluted and from the last enrichment, 0.1 mL of the sample was inoculated onto urea agar plates and then spread using a sterilized L-shaped spreader until the fluid was evenly distributed [21]. The plates were then incubated under aerobic conditions at 30°C for 24 h. Colonies showing urea hydrolyzing potential were purified by subsequent culturing and plating until single bacterial colonies were obtained. Urease production was tested through visual observation of color changes. Thus, isolates with positive ureolytic potential turned the urea agar medium from pale yellow to a pink-red color [29]. From a total of 153 collected colonies, 20 potential urease-producing isolates were selected for further studies.

### 2.3. Quantitative Urease Activity Analysis

For direct assays of urease activity, 1.0 mL of a 24 h old culture was inoculated into bottles containing 9.0 mL of 1.11 M urea solution and monitored for 5 min at 25 ± 2°C. The respective conductivity values were measured and recorded by immersing the probe of the conductivity meter (EC800 Laboratory Benchtop Conductivity Meter, APERA) into the bacterial-urea solution [30]. At the end of the assay, a graph was plotted using conductivity values (ms/cm) against time (min). The rate of conductivity change (ms/cm/min) was acquired from the slope of the plotted graph, which was then multiplied by the dilution factor. This was taken as the ratio of the stock bacteria culture to the sampling bacteria culture before inoculation into the urea solution. The specific urease activity (mM urea hydrolysed/min/OD) was derived by dividing the urease activity (mM urea hydrolysed/min) by the bacterial biomass OD_600_ [31]. The OD was measured using a spectrophotometer (GENESYS™ 20, Thermo Fisher Scientific) at a wavelength of 600 nm:

(1) $$\begin{array}{c} \underset{\left(\text{mM urea hydrolysed}.\min\mathrm{-1}.\text{ΟD}\mathrm{-1}\right)}{\text{specific urease activity}}=\frac{\text{urease activity}\left(\text{mM urea hydrolysed}.\min\mathrm{-1}\right)}{\text{biomass}\left(\text{OD}600\right)}. \end{array}$$

### 2.4. Colony and Cell Morphology

Morphological characterization such as colony and cell morphology; Gram, India ink, and malachite green stain reaction; and motility tests were performed by standard methods [32]. Microscopic observations were performed under a light microscope (Zeiss Axio Lab.A1, Carl Zeiss, with AxioCam Mrm camera) [33].

### 2.5. 16S RNA Gene Amplification

Genomic DNA of each bacterial isolate was extracted using the freeze and thaw protocol and used as a template in a PCR using the primers 8f (5′-AGAGTTTGATCCTGGCTCAG-3′) and 1492r (5′-GGTTACCTTGTTACGACTT-3′) as previously described in [34]. Colonies of overnight grown isolates were picked using a sterilized pipette tip, mixed with 10 *μ*L of PCR grade water in a sterile PCR tube, and placed in a thermocycler with freeze-thaw cycles consisting of three stages with 96°C for 15 min, 90 sec, and 60 sec followed by 15°C for 90 sec at each stage. One microliter of the lysed cells was transferred into 20 *μ*L of PCR master mix. The master mix consists of 16.2 *μ*L PCR grade water, 2 *μ*L of 10x PCR buffer (Life Technologies), 0.4 *μ*L of 10 mM DNTP mix (Life Technologies), 0.4 *μ*L of 20 mg/mL BSA, 0.8 *μ*L of 25 mM MgCl_2_, 0.08 *μ*L of 50 *μ*M of each primer 8f, 1492r, and dream taq-Polymerase (Life Technologies). DNA amplification was performed using a Thermocycler (Verti Cycler, Applied Biosystems).

### 2.6. Nucleotide Sequencing and Analysis

Sequencing was done using the Illumina sequencing facility and the raw DNA chromatogram sequences were viewed and edited using the BioEdit Programme [35] and stored in FASTA format. The forward and reverse sequencing products were assembled using MEGA X after removing poor-quality sequences from the 3′ and 5′ sequence ends. The sequences were blasted against existing sequences in the National Centre for Biotechnological Information (NCBI) database using the Basic Local Alignment Search Tool (BLAST) nucleotide collection database program to search for the closest best match sequence [36].

### 2.7. Optimization of Growth Conditions

The one-factor-at-a-time (OVAT) technique was employed to study the effects of culture conditions affecting bacterial growth such as incubation temperature (4–45°C), initial medium pH (4.0–10.0), and NaCl concentrations (0–20%) in triplicate under oxic conditions. The bacterial cultures were grown in a urea broth base medium (24.0 g/L, HiMedia Laboratories Pvt. Ltd.) and supplemented with filtered urea (5% w/v, Thermo Fisher Scientific) using a 0.45 *μ*m sterile syringe filter [3].

#### 2.7.1. Testing for Optimum pH

To test the range and optimum pH, a medium was prepared at different pH (pH = 3–10) at 0.5 pH intervals. The pH within a desired range 10 mM of the following buffers was maintained. MES buffer (2-(*N*-morpholino)ethanesulfonic acid) was used for the pH range from 3.0 to 6.7; HEPES buffer (4-(2-hydroxyethyl)-1-piperazineethanesulfonic acid) for the pH range from 6.8 to 8.2, HEPPS buffer (3-[4-(2-Hydroxyethyl)piperazin-1-yl]propane-1-sulfonic acid) for the range from 7.3 to 8.7; and CHES buffer (*N*-Cyclohexyl-2-aminoethanesulfonic acid) for the pH range from 8.6 to 10 [37].

For each pH step, 4.5 mL of medium was added to test tubes (triplicate) and inoculated with 0.5 mL of fresh culture. Samples were incubated at 33°C under oxic condition and OD_600_ was recorded at intervals of 0 h to 24 h. The sterile medium was used as blank. Finally, growth curves were plotted as LogOD versus time for each pH, and the optimum was determined. Optimal growth was defined as ≥75% of the highest growth rate achieved [38].

#### 2.7.2. Testing for Optimum Temperature

To test the range and optima temperature, media was prepared at the optimum pH as indicated above. For each temperature, 4.5 mL of medium was added to test tubes (triplicate) and inoculated with 0.5 mL of fresh culture. The tubes were incubated under the oxic condition at temperatures between 5 and 45°C at intervals of 5°C. OD_600_ was recorded at intervals of 1 h for 24 h. The uninoculated medium was used as blank. Finally, the growth curve was plotted as time versus log OD for each temperature and the optimum was determined [39].

#### 2.7.3. Testing for Optimum Salinity

Media were prepared with the optimum pH and for each NaCl concentration to be tested (0, 0.25, 0.5, 1.0, 2.0, 3.0, 4.0, and 5.0 g/L final concentration (w/v)); then, 4.5 mL of medium was added to test tubes (triplicate) and inoculated with 0.5 mL of fresh culture. Tubes were incubated and recorded as indicated above.

### 2.8. Exoenzyme Analysis

Indole formation, aesculin degradation, urease activity, and further exoenzyme activities were determined by using the API ZYM and API20NE test systems (bioMerieux) following the instructions of the manufacturer. API ZYM is a semiquantitative micromethod designed for the research of enzymatic activities [40]. It allows the systematic and rapid study of 19 enzymatic reactions using very small sample quantities [41]. After inoculation, the reaction mixture was incubated for 4–4.5 h at 35°C (optimum temperature) [42]; then, the data was recorded and interpreted.

### 2.9. Phylogenetic Analysis

A phylogenetic tree based on 16S rRNA gene sequences was reconstructed using MEGA version 10.0 [43]. Prior to phylogenetic analysis, primer sequences at both ends were removed and the gaps were adjusted to improve the alignment. Nucleotide sequence alignments were inspected visually to identify positions of uncertain alignments to be corrected or omitted for further analysis [44]. Multiple sequence alignments were obtained using the Clustal-W alignment tool from the MEGA-X software with distance options according to the Kimura two-parameter model and clustering with the maximum likelihood statistical method [44]. Bootstrap analysis based on 1000 replications was used to estimate the confidence level of the tree topologies [43].

## 3. Results

### 3.1. Isolation, Urease Activity, Phylogenetic Analysis, and Morphological Features of the Isolated Bacteria

#### 3.1.1. Isolation and 16S RNA Gene Similarity

Numerous active urease-producing bacterial cultures were enriched and a total of 153 ureolytic pure bacterial colonies were collected after a consecutive restreaking [45]. Twenty strains with high urease activity were identified based on the rapid development of the pink color of the urea agar plates within 24 h of incubation [12] and selected for further investigation. The selected 20 isolates were subjected to partial 16S rRNA gene sequencing [46] at the Leibniz-Institute DSMZ—German Collection of Microorganisms and Cell Cultures. The BLAST results of the sequences searched against the GenBank database using the BLASTN program [47] are summarized in Table 1.

The phylogenetic definition of a species generally would include strains with approximately 70% or greater DNA-DNA relatedness and with 5°C or less ΔT_m_ [48]. A 16S rRNA gene sequence similarity of 98.6% [49] was generally used as a threshold value for species definition in prokaryotes taxonomy. Accordingly, in this study, the sequence analysis showed that 3 (15%), 3 (15%), and 14 (70%) of the isolates belong to the genera *Bacillus, Citrobacter*, and *Enterobacter*, respectively (Table 1).

#### 3.1.2. Specific Urease Activity Testing

**Figure 1:**
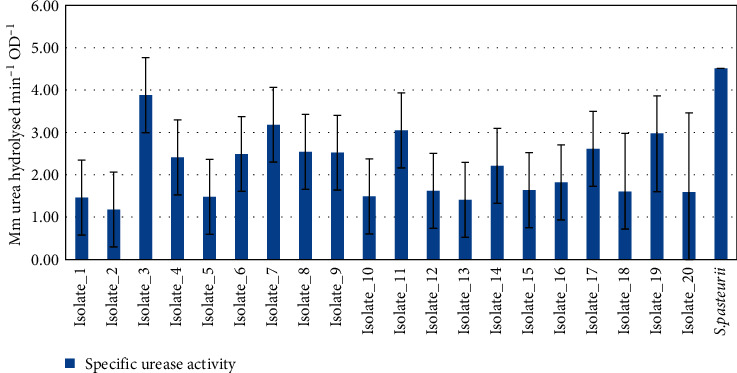
Specific urease activity.

The specific urease activity of each bacterial isolate was measured and the analysis is presented in Figure 1. Based on the quantitative analysis, Isolate_3, Isolate_7, and Isolate_11 showed clearly higher specific urease activity values of 3.88, 3.18, and 3.05 mM urea hydrolysed/min/OD, respectively (*p* < 0.001), and were selected for further analysis. Only these three isolates with higher specific urease activity were selected for analysis due to the limited budget and time during the study time.

#### 3.1.3. Phylogenetic Analysis of the Selected Isolates

**Figure 2:**
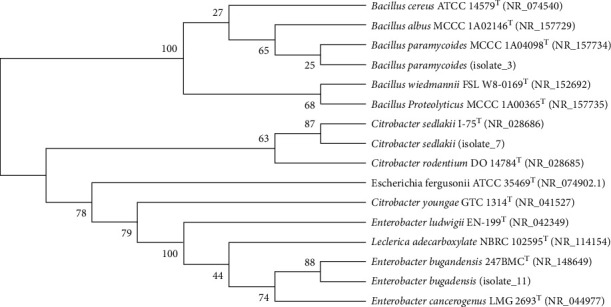
Molecular phylogenetic analysis by maximum likelihood method based on almost-full-length 16S rRNA gene sequences illustrating the phylogenetic position of Isolate_3, Isolate_7, and Isolate_11 and related taxa. The percentage of trees in which the associated taxa clustered together are shown next to the branches.

The phylogeny of the isolates was analyzed using the maximum likelihood method and the Kimura 2-parameter model and included bootstrap analysis based on 1000 replications [43] to estimate the confidence level of the tree topology. The analysis revealed that Isolate_3 was affiliated with the genus *Bacillus* (Figure 2). The highest 16S rRNA gene sequence identity for Isolate_3 (MW723439) was 98.9% and was determined for *Bacillus paramycoides* MCCC 1A04098^T^. The analysis placed Isolate_7 (MW722959) in the vicinity of *Citrobacter sedlakii* I-75^T^ and within the same group (99.2% 16S rRNA sequence identity). The phylogenetic analysis also showed that Isolate_7 is more closely related to Isolate_11 than Isolate_3. Isolate_11 (MW722969) was placed in the neighborhood of *Enterobacter bugandensis* 247BMC^T^ which had a 16S rRNA gene sequence identity of 99.0% to this type of strain.

#### 3.1.4. Cellular and Colony Features of the Selected Isolates

**Figure 3:**
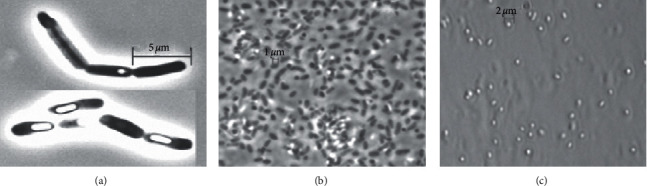
(a, b, c) Phase-contrast photomicrographs of strains: Isolate_3, Isolate_7, and Isolate_11.

Microscopic examination of Isolate_11 showed that cells stained Gram-negative and are single coccobacilli to rod-shaped with an average length of 0.6–1.8 *μ*m (Figure 3(c)). The cells were motile when they were observed in a wet mount with phase-contrast microscopy and by using semisolid agar stabs (agar, 2 mg/L) [50]. Capsules and endospores were not observed after staining with India ink and malachite green, respectively. After incubation at 35°C for 18 h on a nutrient agar medium, the colonies had an average size of 2 mm in diameter and were whitish, smooth, shiny, circular, and convex with entire margins. Colony and microscopic features of Isolate_11 were similar to the recently described strain of *Enterobacter bugandensis* EB-247^T^ [51]. Cells of Isolate_7 stained Gram-negative and were coccobacilli to roads with an average length of 1.6 *μ*m, Gram-negative, nonspore forming, and noncapsulated and occur as single cells or in short chains (Figure 3(b)). After incubation at 35°C for 18 h on nutrient agar plates, colonies were whitish to gray, convex, and circular with an average size of 2.5 mm. These features were similar to the pathogenic *Citrobacter sedlakii* isolated from infant brain samples and grown on sheep blood agar plates [52].

Cells of Isolate_3 were long rods, with an average length of 1.5–4.5 *μ*m and formed highly refractile endospores (Figure 3(a)). Consistent with all other characterized members of the genus *Bacillus*, the cells of Isolate_3 stained Gram-positive [53]. Staining with India ink demonstrated the presence of capsules. Colonies of Isolate_3 were whitish, rough, circular, and nontranslucent and had a rough surface and entire margins, with 1.5–3.5 mm in diameter after incubation at 35°C for 24 h on nutrient agar plates.

### 3.2. Optimum and Range of Growth for pH, Temperature, and Salinity

**Figure 4:**
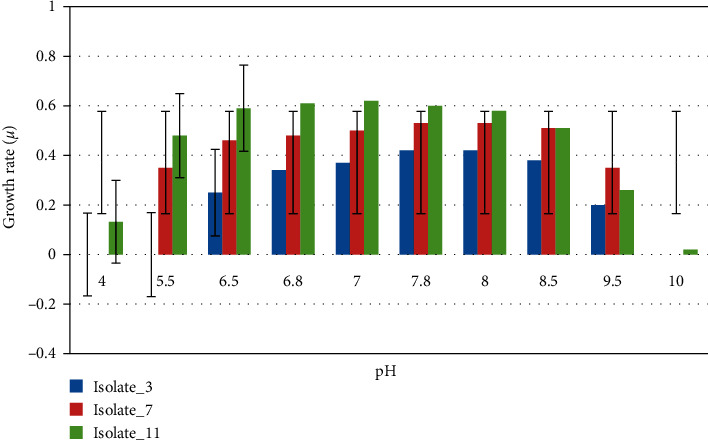
pH growth optimum and range analysis of the strains Isolate_11, Isolate_7, and Isolate_3, respectively.

The pH tolerance analysis showed that Isolate_11 was able to grow in a wider range of pH (pH 4.0–10.0). The optimum growth defined as ≥75% of the highest growth rate achieved [39] was recorded at pH = 5.5–8.0. The highest rate of growth (100%) was recorded at pH = 7.0 (*p* < 0.05) and the lowest rate was at pH = 10.0. Isolate_7 was able to grow between pH = 5.5 and pH = 9.5 and optimum growth was recorded between pH = 6.5 and pH = 8.0. The highest rate of growth (100%) was observed at pH = 8.0 and it was unable to grow at lower pH = 4.0 and higher pH = 10.0. Growth was observed for Isolate_3 between pH of 6.5 and pH of 9.5; optimal growth (≥75%) was recorded between pH of 7.0 and pH of 8.0 (*p* < 0.05); and the highest rate of growth (100%) was recorded at pH = 7.4. Isolate_3 was unable to grow or showed limited growth between pH = 2.8 and pH = 5.0 (Figure 4).

**Figure 5:**
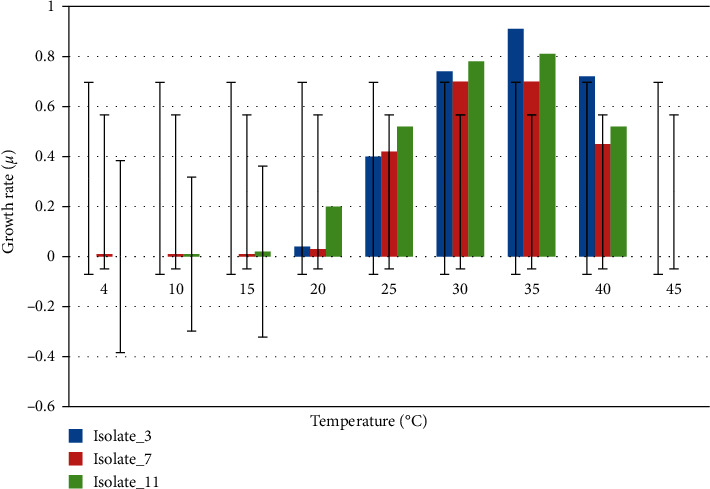
Temperature optimum and range analysis of strains of Isolates_11, Isolate_7, and Isolate_3, respectively.

The analysis of the OD values after 24 h of incubation at different temperatures showed that Isolate_11 could grow between 20°C and 40°C with both optimum growth (≥75% of the highest growth rate) and maximum growth rate (100%) at 35°C (*p* < 0.05). It was unable to grow at a temperature range of 4–15°C. Similarly, Isolate_7 was able to grow between 20°C and 40°C (optimum at 25°C–40°C), with the highest rate of growth (100%) at 30°C and 35°C (*p* < 0.05). It showed no or very slow growth rate between 4°C–25°C and 45°C. Isolate_3 was able to grow between 25°C and 40°C with optimum growth between 35°C and 40°C. It showed (very) limited or no growth between 4°C–20°C and 45°C (Figure 5).

**Figure 6:**
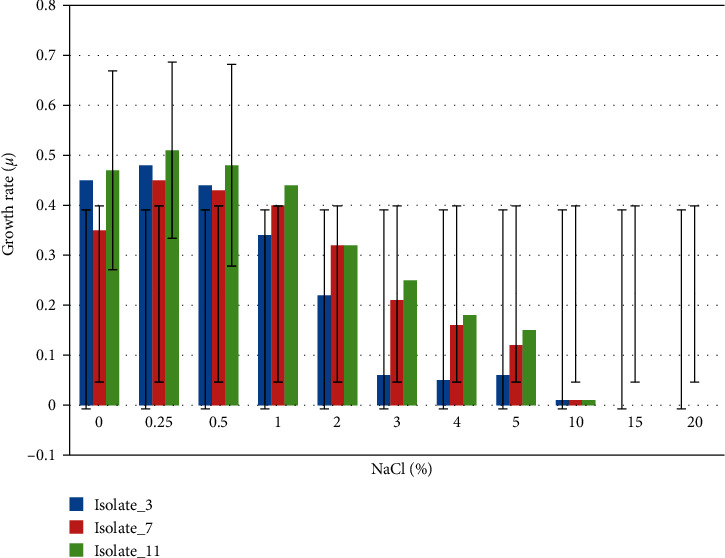
NaCl tolerance, optimum, and range analysis of the strains of Isolate_7, Isolate_11, and Isolate_3, respectively.

The study of NaCl concentration tolerance also revealed that Isolate_11 was able to grow in NaCl concentration range of 0.0–5.0%, where it grew best (optimum growth) between 0.25 and 3.0% (w/v) in 48 h of incubation; the highest rate of growth (100%) was recorded at 0.25%. During the study, it was observed that Isolate_11 was unable to grow at NaCl concentrations ≥10%. Optimal growth was observed between 0.0 and 2.0% (w/v) NaCl for Isolate_7 and between 0.0 and 0.5% NaCl for Isolate_3 after 24 h of incubation. The highest rate of growth was recorded at 0.25% NaCl for both strains and they showed zero growth at NaCl concentrations of ≥10% (Figure 6).

### 3.3. Exoenzyme Profiles of the Selected Strains

In addition to urease activity, the selected isolates showed activities for various exoenzymes (Table 2). Out of 25 tested exoenzymes, Isolate_11 showed activities for 14 exoenzymes; Isolate_7 showed for 13 exoenzymes; and Isolate_3 for 11 exoenzymes. All the three selected strains showed similar preferences towards phosphate-containing compounds (alkaline phosphatase, acid phosphatase, and naphthol-AS-BI-phosphohydrolase), peptidase activities (leucine arylamidase and valine arylamidase), and nitrate reductase activity. They all assimilated N-acetyl-glucosamine, D-maltose, potassium gluconate, and trisodium citrate. Isolate_3 and Isolate_7 showed a similar positive reaction for lipids (esterase and esterase lipase), while Isolate_11 and Isolate_7 showed similar preferences for trypsin and *β*-galactosidase. Isolate_11 showed unique preferences for various sugars (*α*-glucosidase, *β*-glucosidase, and N-acetyl-*β*-glucosaminidase).

## 4. Discussions

This study was conducted with the aim of isolation and characterization of rapid urease-producing bacteria from Ethiopian soils. In the study, twenty urease-producing bacterial isolates were identified using a qualitative urease activity assay. Among these, three of them (*Bacillus paramycoides*, *Citrobacter sedlakii*, and *Enterobacter bugandensis*) expressed urease at high rates (3.88, 3.18, and 3.05 of mM urea hydrolysed min^−1^ OD^−1^) (*p* < 0.05) as determined by a conductivity assay. Literature showed that urease was studied from several bacterial strains such as *Bacillus* [54], *Citrobacter*, *Enterobacter*, *Pseudomonas*, *Serratia*, and *Yersinia* [55]. In our study, the identified strains exhibiting urease activity were identified as belonging to the genera *Bacillus*, *Citrobacter*, and *Enterobacter*.

Consistent with previous studies done on *Bacillus paramycoides* MCCC 1A04098^T^*, Citrobacter sedlakii* 2596^T^ and *Enterobacter bugandensis* EB-261^T^ [51, 52, 56], similar morphological and physiological characteristics were observed with Isolate_3, Isolate_7, and Isolate_11, respectively, and later confirmed by 16S rRNA gene sequencing. However, morphological studies have revealed that Isolate_3 was a spore former with conspicuous spore and had a rough colony and cells having a length of 1.5–4.5 *μ*m after incubation at 35°C for 18 h. This makes Isolate_3 different from the previously characterized novel strain of *B. paramycoides* MCCC 1A04098^T^ which was reported as nonspore forming, with 1.8–2.2 *μ*m in length and with waxy colonies after incubation at 32°C for 48 h on LB medium [56]. Besides, unlike the nonureolytic strain of *B. paramycoides* MCCC 1A04098^T^, Isolate_3 is a urease producer. These are important key characteristics that differentiate our strain from the previously identified related strain of *B. paramycoides* MCCC 1A04098^T^. Therefore, it is significant to note that Isolate_3 showed unique morphological and physiological features. On this basis, the isolate described here probably represents a new member of the genus *Bacillus*.

In addition, the morphological and physiological studies of Isolate_11 showed different characters from the previously characterized nonureolytic *E. bugandensis* strain EB-247^T^ [51], as Isolate_11 does not form a capsule but secretes urease and gelatinase enzymes and assimilates D-arabinose following incubation for 18 h at 35°C, while the former was incubated for 24 h at 37°C on MacConkey agar. The most prominent biochemical feature of Isolate_7 was its ability to assimilate aesculin ferric citrate, which agrees with the name of the genus (*Citrobacter* = citrate utilizing rods) [57]. Furthermore, similar biochemical and morphological features were also observed between Isolate_7 and clinical isolate *Citrobacter sedlakii* 2596^T^ [52] with respect to urease activity, arginine hydrolase, and fermentation of arabinose, mannitol, and maltose. Jacob [57] also reported a similar positive reaction for urease, arginase, lipase, and *β*-glucosidase for halophilic *Citrobacter* strains isolated from the saline environment. But, the reported strain had filamentous and rough colonies unlike Isolate_7, which showed convex and circular colonies with an average size of 2.5 mm. Brenner et al. [58] reported that *Citrobacter sedlakii* ATCC 51118^T^ has similar positive results to Isolate_7 with respect to urease activity and arginine dihydrolase and is different in its negative reaction with aesculin substrate utilization.

Though the selected strains were isolated from mountainous agricultural land termite mound soil (Isolate_11) with a slightly neutral pH of 6.7 and rift valley grassland termite mound soil (Isolate_3 and Isolate_7) with slightly alkaline pH, respectively, they tolerated a broader pH range as explained above and based on their optimum growth pH of 5.5–8.5 (*p* < 0.05), they are categorized as neutrophiles [59]. They were grown at moderate temperatures between 20°C and 40°C and with an optimum growth temperature in the range of 30–39°C. Therefore, they are categorized as mesophilic bacteria [60]. The isolates showed a narrow tolerance range for temperature corresponding to their origin from Ethiopian soil.

As shown in Figure 5, all the three selected strains tolerated NaCl concentrations of up to 5% (w/v), which exceeds the maximum NaCl tolerance of common soil bacteria. This increased NaCl tolerance constitutes an important differential characteristic of the selected species. It best explains the higher salinity in Ethiopian soils [61], which could be due to heavy fertilizer application, use of poor quality irrigation water, and inadequate drainage [64, 65, 66]. Nonhalophiles grow optimally at less than 2% NaCl; slight halophiles grow optimally at 2–5% NaCl; moderate halophiles grow optimally at 5–20% NaCl; and extreme halophiles grow optimally above 20–30% NaCl [62]. This implies that the rapid urease-producing isolates in this study could be considered as nonhalophilic to slightly halophilic bacteria. Further studies of such halophilic bacteria could help to discover new enzymes to be applied in biocatalytic processes that are faster, more accurate, specific, and environmentally friendly [63]. These enzymes could keep high activity and stability in salty environments and could have potential application values in agriculture, engineering, and medicine.

## 5. Conclusions

The results obtained from this research confirmed the presence of ureolytic bacteria in Ethiopian soil indicating their adaptation from the rift valley to mountainous ecosystems of the country. In the study, new strains of *Bacillus*, *Citrobacter*, and *Enterobacter* were isolated from Ethiopian soil and characterized based on their distinctive physiological and morphological characteristics. From the study, it was shown that the three isolates (Isolate_3, Isolate_7, and Isolate_11) had relatively more rapid rates of urea hydrolysis and were found to be nonhalophilic to slightly halophilic neutrophiles and aerobic mesophiles with a range of tolerance towards pH (4.0–10.0), NaCl (0.25–5%), and temperature (20–40°C). Further studies on the growth profiles of the isolates, calcite precipitation, soil biocementation, and scanning electron microscopy analysis were recommended for future studies.

## Figures and Tables

**Table 1:** 16S rRNA genes sequence similarity with the closest strains of the isolates.

Code	Closest strain	%	Origin
Isolate_1	*Bacillus paramycoides* MCCC 1A04098^T^	99.6	Tulubolo
Isolate_2	*Enterobacter tabaci* YIM Hb-3^T^	97.1	Lake Chitu
Isolate_3 (MW723439)	*Bacillus paramycoides* MCCC 1A04098^T^	98.9	West Wellega
Isolate_4	*Enterobacter tabaci* YIM Hb-3^T^	97.9	Lake Chitu
Isolate_5	*Enterobacter asburiae* JCM 6051^T^	99.0	Wonji
Isolate_6	*Enterobacter tabaci* YIM Hb-3^T^	98.9	Lake Chitu
Isolate_7 (MW722959)	*Citrobacter sedlakii* I-25^T^	99.8	Wonji
Isolate_8	*Citrobacter sedlakii* I-25^T^	99.6	Lake Chitu
Isolate_9	*Enterobacter tabaci* YIM Hb-3^T^	98.9	Lake Chitu
Isolate_10	Enterobacter hormaechei subsp. *hormaechei* 10–17^T^	98.0	Yabello
Isolate_11 (MW 722969)	*Enterobacter bugandensis* 247BMC	99.2	Wonji
Isolate_12	Enterobacter hormaechei subsp. *hormaechei* 10–17^T^	99.9	Lake Abijata
Isolate_13	*Enterobacter tabaci* YIM Hb-3^T^	98.2	Lake Abijata
Isolate_14	*Bacillus wiedmannii* FSLW8-0169^T^	98.7	Tulubolo
Isolate_15	*Enterobacter tabaci* YIM Hb-3^T^	98.9	West Wellega
Isolate_16	*Enterobacter tabaci* YIM Hb-3^T^	99.0	Yabello
Isolate_17	*Enterobacter asburiae* JCM 6051^T^	97.2	Yabello
Isolate_18	*Citrobacter sedlakii* I-25^T^	99.2	Tulubolo
Isolate_19	*Enterobacter tabaci* YIM Hb-3^T^	98.2	Tulubolo
Isolate_20	*Enterobacter tabaci* YIM Hb-3^T^	98.8	Yabello

% indicates similarity.

**Table 2:** Biochemical and exoenzyme profiles of the three selected strains.

Characteristics	Isolate_3	Isolate_7	Isolate_11
Control	−	−	−
API 20NE			
Nitrate reductase	+	+	+
Indole formation	−	+	−
Arginine dihydrolase	−	W	+
Urease	+	+	+
Protease	+	−	+
L-Arabinose	−	+	+
API ZYM			
Alkaline phosphatase	+	+	+
Esterase (C4)	W	W	−
Esterase lipase (C8)	W	W	−
Lipase (C14)	−	−	−
Leucine arylamidase	+	+	+
Valine arylamidase	W	W	+
Cystine arylamidase	−	−	−
Trypsin	−	W	W
*α*-Chymotrypsin	+	−	−
Acid phosphatase	+	+	+
Naphtanol-AS-BI_phosphohydrolase	W	W	W
*α*-Galactosidase	−	−	−
*β*-Galactosidase	−	+	+
*β*-Glucuronidase	−	−	−
*α*-Glucosidase	+	−	W
*β*-Glucosidase	−	−	W
N-Acetyl-*β*-glucosaminidase	−	−	W
*α*-Mannosidase	−	−	−
*α*-Fucosidase	−	−	−

“+” = positive; “W” = weakly positive; and “−” = negative.

## Data Availability

The data are available upon request to the corresponding author.
